# A preliminary animal study on the prediction of nerve block success using ultrasonographic parameters

**DOI:** 10.1038/s41598-022-06986-y

**Published:** 2022-02-24

**Authors:** Emiko Chiba, Kohei Hamamoto, Eiichi Kanai, Noriko Oyama-Manabe, Kiyoka Omoto

**Affiliations:** 1 Department of Radiology, Jichi Medical University Saitama Medical Center, 1-847 Amanuma-cho, Omiya-ku, Saitama, Saitama 330-8503 Japan; 2 Laboratory of Small Animal Surgery, Department of Veterinary Medicine, Azabu University, 1-17-71, Fuchinobe, Chuo-ku, Sagamihara, Kanagawa 252-5201 Japan; 3 Department of Laboratory Medicine, Jichi Medical University Saitama Medical Center, 1-847 Amanuma-cho, Omiya-ku, Saitama, Saitama 330-8503 Japan

**Keywords:** Diagnostic markers, Predictive markers, Preclinical research

## Abstract

This study aimed to evaluate the diagnostic value of ultrasonographic parameters as an indicator for predicting regional nerve block success. Ultrasound-guided sciatic nerve block was performed in seven dogs using either 2% mepivacaine (nerve-block group) or saline (sham-block group). The cross-sectional area (CSA), nerve blood flow (NBF), and shear wave velocity (SWV) of the sciatic nerve (SWV_N_), SWV of the biceps femoris muscle (SWV_M_), and their ratio (SWV_NMR_) were measured at 0, 30, 60, and 90 min after the nerve block as well as the change rate of each parameter from the baseline. A receiver operating characteristic (ROC) curve analysis was performed to determine the diagnostic value of each parameter in the prediction of nerve block success. No significant changes were observed in the CSA or NBF in association with the nerve block. The SWV_N_ and SWV_NMR_ in the nerve-block group were significantly higher than those in the sham-block group at 90 min and at 30, 60, and 90 min, respectively (*p* < 0.05). The change rates of SWV_N_ and SWV_NMR_ in the nerve-block group were significantly higher than those in the sham-block group at all time points (*p* < 0.05). The ROC curve analysis showed that SWV_N_ had a moderate diagnostic accuracy (area under the curve [AUC], 0.779), whereas SWV_NMR_ and change rates of SWV_N_ and SWV_NMR_ had a high diagnostic accuracy (AUC, 0.947, 0.998, and 1.000, respectively). Ultrasonographic evaluation of the SWV_N_ and SWV_NMR_ could be used as indicators for predicting nerve block success.

## Introduction

An ultrasound (US)-guided nerve block is a type of regional anesthesia used for periprocedural analgesia in various medical fields such as orthopedics and endovascular treatment^1–7^. Since the nerve block technique requires ample time to elicit the required analgesic effect and its success rate is approximately 90%^8–11^, it is important to determine the success of a nerve block before a painful procedure is initiated. Several skin sensation tests, such as the light-touch test, pinprick test, and cold-sensation test, are commonly used to measure the success of a nerve block, but these methods are subjective and sometimes fail to do so^12,13^. An electrical stimulation test can be used as an alternative, objective method, but it is invasive. In addition, in patients under general anesthesia, testing modalities that rely on conscious cooperation are no longer possible. Therefore, to determine the success of nerve blocks more accurately and easily, a novel evaluation method that is objective, quantitative, and noninvasive should be developed.

Ultrasonography is widely accepted as a valuable diagnostic tool for peripheral nerve disorders^14–24^. Studies on the utility of ultrasonography for peripheral nerve disorders initially focused on the evaluation of compressive neuropathies such as carpal tunnel syndrome, cubital tunnel syndrome, and fibular neuropathy. Several features revealed by B-mode ultrasound and color Doppler imaging have been examined in the evaluation of compressive neuropathies, including an increase in the nerve cross-sectional area (CSA) proximal to the site of nerve compression, loss of normal fascicular architecture, reduced or excessive nerve mobility, and increased vascularity^17,22–24^. Ultrasound elastography has emerged as a noninvasive tool for the evaluation of nerve stiffness. This technique estimates tissue elasticity as a physical property termed as the Young’s modulus, which is a proportionality constant that relates applied force per unit area or stress with the resultant relative change in tissue dimension or strain. Several clinical studies that have used shear wave elastography (SWE) to evaluate the median nerve in carpal tunnel syndrome and the tibial nerve in diabetic neuropathy have reported that the SWE modality can depict an increase in nerve stiffness in a noninvasive manner^15,16,19–21,25^. In addition, the utility of a multiparametric ultrasonographic assessment including SWE has been reported not only for the evaluation of the pathology of nerves but also for the quantitative evaluation of muscular atrophy secondary to nerve injury^26,27^.

Regional nerve blocks with local anesthetics induce various physiological changes, including a decrease in muscular tone, an increase in blood flow, and an increase in skin temperature^28–31^. Additionally, local anesthetic agents have been shown to induce a reduction in nerve blood flow^32,33^. Based on these findings, it seems possible that an ultrasonographic assessment of the nerve, muscle, and blood flow could identify such changes after a nerve block and that such an assessment could be a novel indicator for determining nerve block success. However, this possibility has not been examined in a previous study.

This study aimed to (1) assess the changes in various ultrasonographic parameters including CSA, intraneural blood flow, and SWEs in the nerve and muscle in association with a regional nerve block and (2) evaluate the diagnostic value of these ultrasonographic parameters as an indicator for predicting regional nerve block success.

## Materials and methods

### Animals

All experimental protocols of this study were approved by the Animal Research Committee of the Azabu University and all methods were carried out in accordance with the Guide for the Animal Care and Use Committee at Azabu University. The authors have read the ARRIVE guidelines and the study was carried out in compliance with the ARRIVE guidelines. Seven healthy beagles (three sexually intact females and four sexually intact males) were included in the study. The dogs were between 1 and 3 years of age, and weighed between 8 and 13 kg. In each dog, the nerve block was performed using an anesthetic agent in one hind limb and normal saline in the other hind limb, as described below, and the respective legs were categorized into the nerve-block group and sham-block group simulating a failed nerve block. All procedures were performed under general anesthesia. After subcutaneous administration of atropine (0.05 mg/kg) (Fuso Pharmaceutical Industries Ltd., Osaka, Japan) for preanesthetic medication, anesthesia was induced by intravenous administration of propofol 0.6–0.8 ml/kg (Maruishi Pharmaceutical Co., Ltd., Osaka, Japan). The dog was intubated with a cuffed endotracheal tube and mechanically ventilated with volume-controlled ventilation. Anesthesia was maintained using 1–3% isoflurane. The monitoring included a limb-lead electrocardiogram and pulse oximetry. An esophageal temperature probe and heating pad were used to maintain the dog's body temperature at > 37.5 °C.

### US equipment

All US images were acquired using a commercially available US unit (Aplio 300, Canon Medical Systems, Tochigi, Japan). The transducers and imaging parameters for US-guided nerve block and each US measurement are summarized in Table 1.

**Table 1:** The transducers and imaging parameters for US-guided nerve block and US measurements.

	Transducers	Imaging parameters
US-guided nerve block	13–18 MHz linear array probe (PLT-1005BT)	Probe frequency, 18 MHz; optimal image setting for visualization of the sciatic nerve
CSA measurement	13–18 MHz linear array probe (PLT-1005BT)	Probe frequency, 18 MHz; optimal image setting for visualization of the sciatic nerve
Nerve blood flow analysis	13–18 MHz linear array probe (PLT-1005BT)	SMI frequency, 7.2 MHz; flow scale, 1.5 cm per sec; frame rate, 58 frame per sec; dynamic range, 65 dB, color gain 40 dB, pulse repetition frequency, 21.9 kHz, SMI filter, 2
SWE measurement	7.2–14.0 MHz linear array probe (PLT-1204BT)	Probe frequency, 14 MHz; optimal image setting for visualization of the sciatic nerve; display scale of SWV, 0.5–6.5 m/s

*CSA* cross-sectional area, *SMI* superb microvascular imaging, *SWE* shear wave elastography, *SWV* shear wave velocity.

### US-guided sciatic nerve block

**Figure 1:**
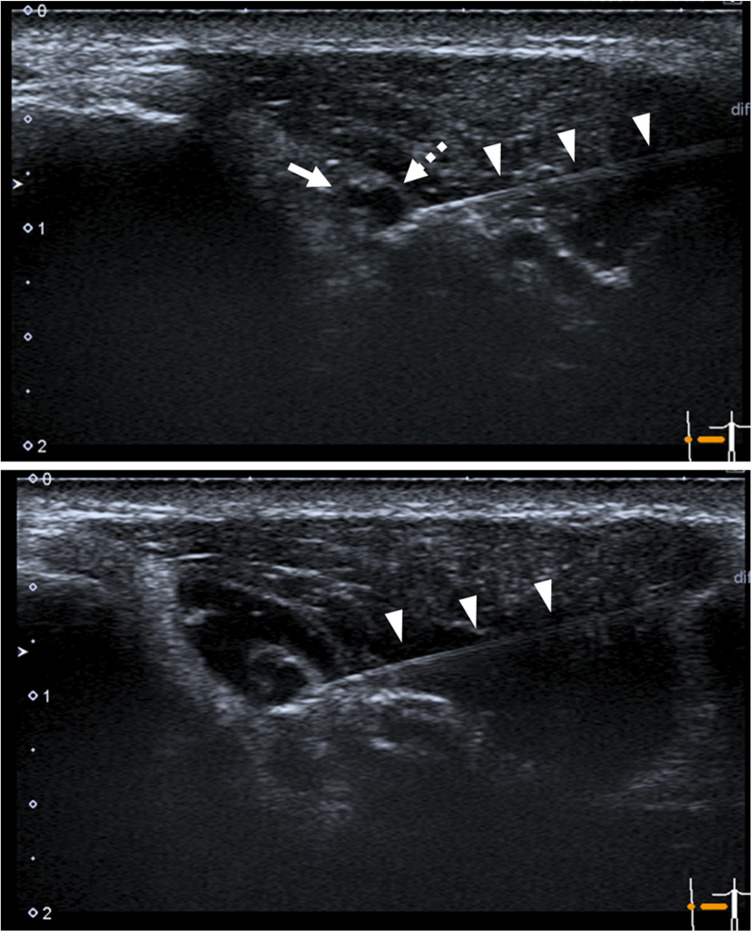
Ultrasound (US) image of US-guided sciatic nerve blocks. Two components of the sciatic nerve, the tibial (*dashed arrow*) and common peroneal nerves (*arrow*), are visualized (*upper panel*). The *arrowheads* indicate the needle trajectory. The injected anesthetic agent is shown as a low echoic area around the tip of the needle (*lower panel*).

US-guided sciatic nerve block was performed according to published techniques^34,35^. Briefly, the US probe was placed perpendicular to the sacrotuberous ligament at a level above the greater trochanter. In this orientation, the sciatic nerve was depicted in a cross-section under the superficial gluteal muscle. At this level, the sciatic nerve was visualized as two components: the tibial nerve and the common peroneal nerve (Fig. 1). A 22-gauge, 50-mm insulated stimulating needle (Stimuplex Ultra 360, B. Braun Aesculap Japan Co., Ltd., Tokyo, Japan) connected to a nerve stimulator (Stimuplex HNS 12, B. Braun Aesculap Japan Co., Ltd.) was slowly advanced toward the sciatic nerve using an in-plane technique guided by ultrasound. Correct needle position was confirmed when foot plantar flexion was maintained at a current of 0.5 mA. Subsequently, 2 ml of 2% mepivacaine (Aspen Japan Co., Ltd., Tokyo, Japan) or the same volume of normal saline was injected around the sciatic nerve in the nerve-block and sham-block legs, respectively (Fig. 1).

### Nerve stimulation test

We used an electrical nerve stimulation test as the standard reference for nerve block success. Nerve stimulation of the sciatic nerve was performed at the level of the greater trochanter of the femur to avoid the effect of fluid collection due to the nerve block. As described above, an insulated stimulating needle, as used in nerve block, was advanced adjacent to the epineurium of the sciatic nerve under US-guidance, and then the nerve stimulator was turned on. The nerve stimulator was set to deliver impulses at 3 Hz with 0.1 and 1.0 ms (SENSe mode) for electrolocation. Correct electrolocation was indicated by dorsiflexion of the foot. Before the nerve block, a muscle contraction caused by a current of < 0.5 mA was considered a normal response in the nerve stimulation test. The lowest current capable of stimulating a muscle twitch and the approximate position of the needle were recorded for each site. After the nerve block, no fluid retention was confirmed by US at the stimulated site, and electrical stimulation was performed at the same position as before the nerve block. A successful block was defined as no muscle contraction caused by stimulation with the same current as that used before the nerve block. All tests were performed by an experienced sonographer blinded to whether the leg was in the nerve-block or sham-block group. All nerve stimulation tests were performed after the completion of US measurements.

### US measurements

All US images were assessed by an experienced sonographer blinded to the results of the nerve stimulation test and grouping. The US images described below were serially collected before the nerve block (0 min) and at 30, 60, and 90 min after the nerve block at a point 5 cm distal from the block point. All measurements were performed before the nerve stimulation test to avoid the influence of electrical stimulation on the measurement values.

### CSA measurement

The cross-sectional area (CSA) of the sciatic nerve was determined from the short-axis view and measured using electronic calipers. Three measurements were taken and averaged for the statistical analysis.

### Nerve blood flow analysis

We measured the microvascular blood flow within the epineurium of the sciatic nerve using superb microvascular imaging (SMI), which enables the accurate visualization of vascular structures with intensive clutter suppression to provide flow signals for large to small vessels, and the SMI presents these data at high frame rates^36^. The SMI was taken from the short-axis view at the same position as the CSA measurements. The SMI parameters are listed in Table 1. The US images were sequentially recorded for 5 s in the same section in the dual-image display (grayscale and monochromatic SMI) mode, and the image with the highest blood flow signal within the epineurium was used for analysis. A semi-quantitative analysis of the SMI signals was performed using Image J software (ver. 1.52, U.S. National Institutes of Health, Bethesda, MD, USA). On the US equipment, the margins of epineurium were manually outlined as regions of interest (ROI) on the grayscale image and automatically outlined at the same position on the SMI image. The data were exported as DICOM files. On Image J, the grayscale pixels within these ROIs of the SMI were calculated, and these data are presented herein as the nerve blood flow (NBF) at arbitrary units. Three measurements were taken and averaged for the statistical analysis.

### SWE measurement

**Figure 2:**
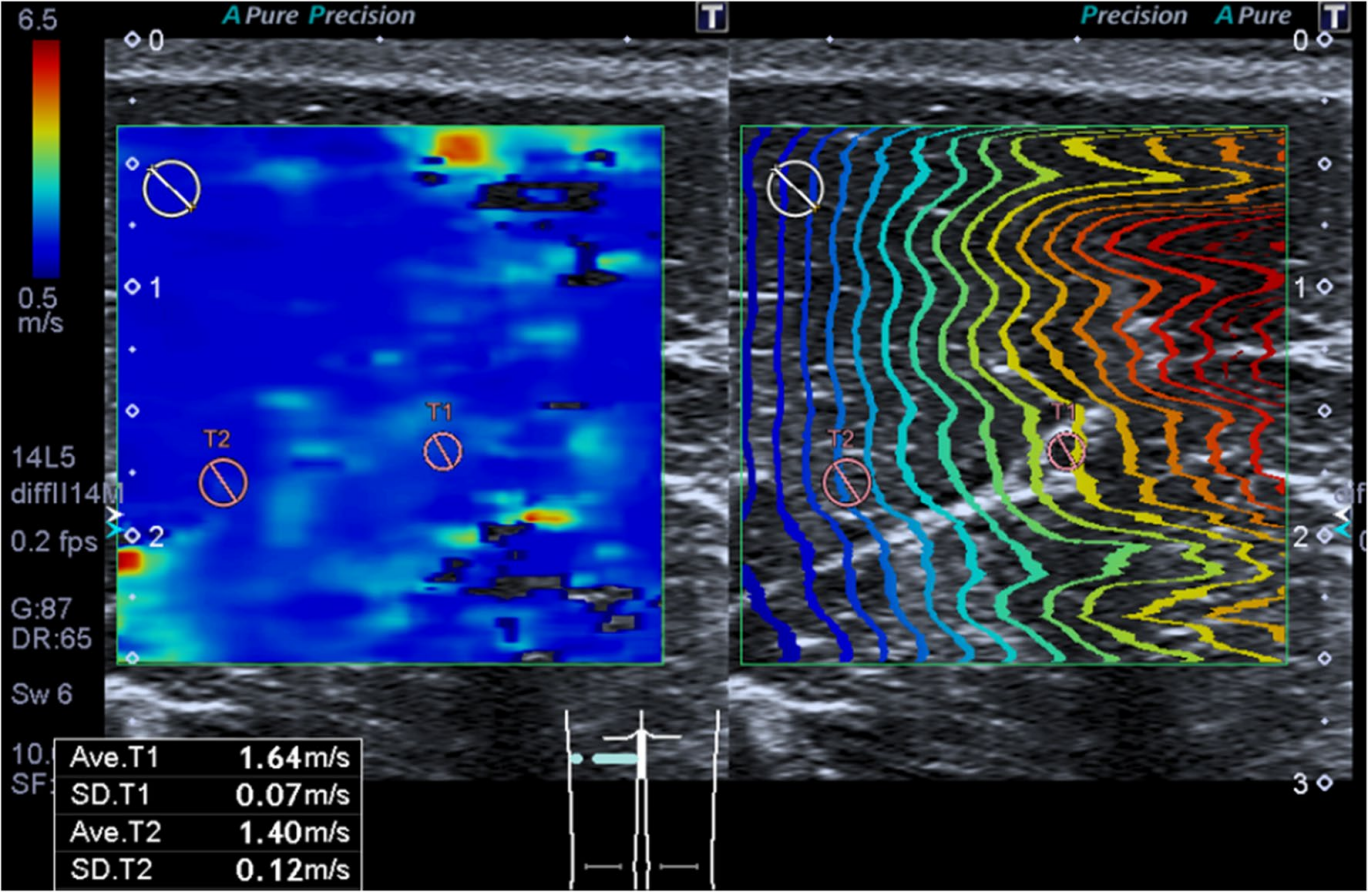
Images of the sciatic nerve and biceps femoris muscle of a dog in the speed mode of shear wave elastography. The images for the speed mode are the shear wave velocity (m/s) map (*left image*) and the map for the propagation mode (*right image*). Note that consistent parallel contour lines are shown in the propagation mode. *Red circles* of T1 (2 mm in diameter) and T2 (diameter: 4 mm) indicate the ROIs of the sciatic nerve and biceps femoris muscle, respectively.

Elastographic examinations were carefully performed to avoid transducer compression. In the present study, we measured the shear wave velocity (SWV) of each sciatic nerve (SWV_N_) and the biceps femoris muscle (SWV_M_) that is innervated by the sciatic nerve. Additionally, the ratio of the nerve and muscle SWV values (SWV_NMR_) at the same measurement was calculated since we hypothesized that this value can indicate the success of the nerve block. The sciatic nerve was identified on the short-axis view at the same position as the CSA and NBF measurements, allowing the visualization of the transverse view of the mid-belly of the biceps femoris muscle. The elasticity mode was initiated by positioning a square ROI including both the sciatic nerve and the muscle. We confirmed the reliability of the data obtained by SWE using the proper propagation mode (Fig. 2). The data reliability is high when the contour lines are nearly straight and regularly parallel to each other and low when they are irregularly distorted and chaotic. We placed 2-mm and 4-mm diameter ROIs on the areas with parallel contour lines in the sciatic nerve and muscle, respectively (Fig. 2). In the sciatic nerve, the ROI was placed on the tibial nerve component because the diameter of the tibial nerve component was larger than that of the peroneal nerve component. At least three validated measurements were performed for each nerve and muscle. The mean SWV values of the sciatic nerve and muscles are presented.

### Evaluation of the change rate of the US parameters during the nerve block

Previous studies demonstrated that US parameters including CSA and tissue elasticity of the peripheral nerve change with various factors including limb position and movement, laterality, and age^34–41^. Therefore, we calculated the change rate in each US parameter from the baseline value (0 min) at each time point (30, 60, and 90 min) after the nerve block in the same dog, in addition to the actual measurement values, to avoid the influence of these differences between individuals in assessing the changes in US parameters associated with a nerve block.

### Statistical analysis

All data are presented as the mean ± standard deviation (SD). The coefficient of variance (CV) was obtained by dividing each SD by the mean. Statistical analyses were performed using a commercially available software program (EZR, ver. 1.53, Saitama, Japan), which is a graphical user interface for R (The R Foundation for Statistical Computing, ver. 4.0.2, Vienna, Austria). More precisely, it is a modified version of R commander designed to add statistical functions frequently used in biostatistics^42^. Differences within the nerve-block and sham-block groups were analyzed using Kruskal–Wallis test. The Steel–Dwass post-hoc test was used to compare all pairs of groups. Wilcoxon rank-sum test was used to compare the parameters between the nerve-block and sham-block groups. A receiver operating characteristic (ROC) curve analysis was performed when the difference in the parameters was significant. Optimal cutoff values for predicting nerve block success were obtained by maximizing the Youden index, and the diagnostic performance of the given values was presented as the sensitivity, specificity, positive predictive value, and negative predictive value. To test the difference between the areas under the ROC curves for each parameter, a comparison of ROC curves was performed using EZR. Differences were considered significant when the *p*-value was < 0.05. We assessed the intra-rater reliability of the three serial measurements of each US parameter by a sonographer as described above using intra-class correlation coefficients^43^ (ICC; 1,1); furthermore, a 95% confidence interval (CI) was set for each ICC. An ICC > 0.80 was indicative of excellent agreement.

## Results

### US-guided sciatic nerve block

The sciatic nerve was easily identified in all seven dogs. The success rate for US-sciatic nerve block was 100%, and there were no major complications. In the nerve-stimulation test, a complete motor block of the sciatic nerve was achieved in the nerve-block group of all dogs at 30 min after the nerve block and was still present at 90 min. In contrast, no motor block was observed at any time point in the sham block group.

### Changes in the US parameters associated with the nerve block

Excellent intra-rater reliability was observed for all US parameters. The results are presented in Table 2.

**Table 2:** Intra-rater reliability of the US parameters.

	Nerve-block group				Sham-block group			
	0 min	30 min	60 min	90 min	0 min	30 min	60 min	90 min
CSA	0.984 (0.945–0.997)	0.981 (0.935–0.996)	0.984 (0.939–0.998)	0.988 (0.9510.998)	0.987 (0.953–0.997)	0.980 (0.930–0.996)	0.984 (0.939–0.998)	0.992 (0.969–0.999)
NBF	0.981 (0.935–0.996)	0.924 (0.732–0.986)	0.985 (0.948–0.997)	0.970 (0.896–0.994)	0.905 (0.668–0.982)	0.966 (0.881–0.994)	0.952 (0.832–0.991)	0.918 (0.714–0.985)
SWV_N_	0.943 (0.799–0.989)	0.983 (0.941–0.997)	0.919 (0.716 0.985)	0.901 (0.654–0.981)	0.940 (0.789–0.989)	0.888 (0.608–0.979)	0.906 (0.670–0.982)	0.923 (0.729–0.985)
SWV_M_	0.838 (0.433–0.969)	0.894 (0.628–0.980)	0.932 (0.61–0.987)	0.803 (0.310–0.963)	0.928 (0.748–0.986)	0.819 (0.365–0.866)	0.878 (0.574–0.977)	0.834 (0.420–0.969)
SWV_NMR_	0.952 (0.830–0.991)	0.888 (0.608–0.979)	0.859 (0.505–0.973)	0.917 (0.709–0.984)	0.955 (0.843–0.992)	0.854 (0.489–0.972)	0.889 (0.613–0.979)	0.876 (0.565–0.977)

Values represent intra-class correlation coefficient (ICC). Numbers in parentheses are 95% confidence Intervals. ICC (1,1) was used for intra-rater reliability.

*CSA* cross-sectional area, *NBF* nerve blood flow, *SWV*_*N*_ shear wave velocity of the sciatic nerve, *SWV*_*M*_ shear wave velocity of the muscle, *SWV*_*NMR*_ the ratio of shear wave velocity of the nerve to muscle.

**Figure 3:**
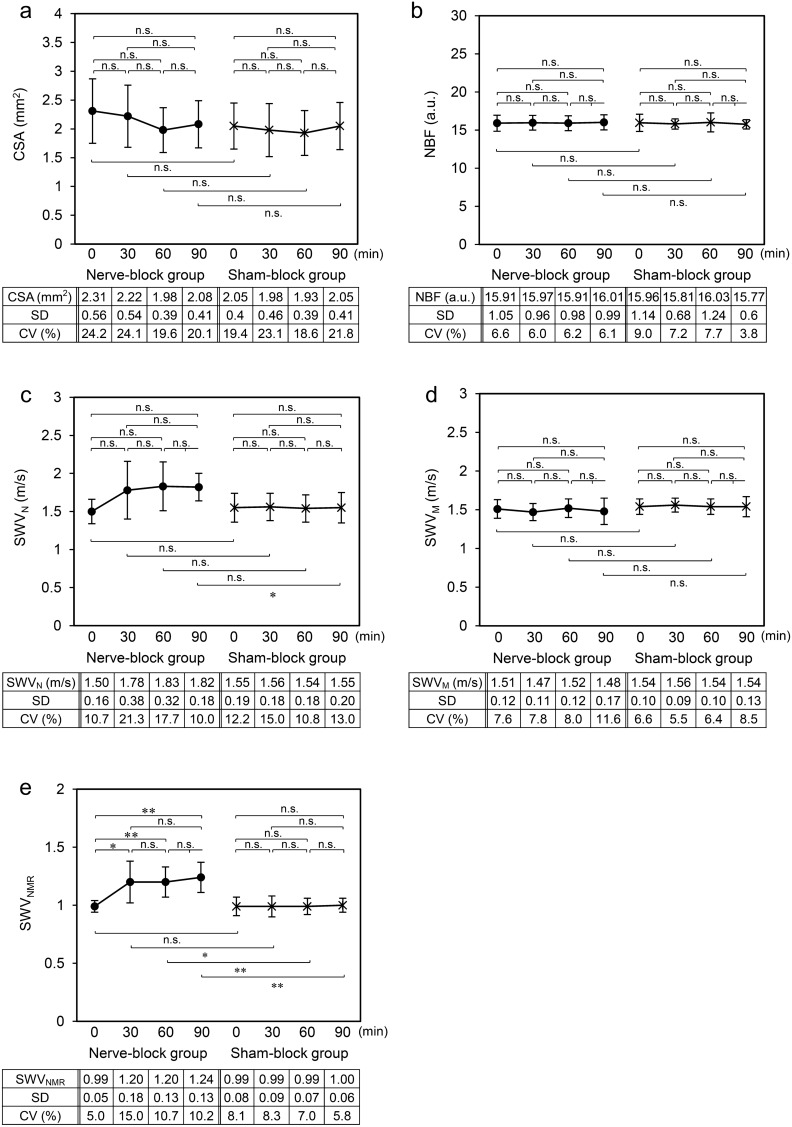
The change in the US parameters associated with the nerve block. (**a**) Cross-sectional area (CSA). (**b**) Nerve blood flow (NBF). (**c**) Shear wave velocity of the sciatic nerve (SWV_N_). (**d**) Shear wave velocity of the muscle (SWV_M_). (**e**) The ratio of shear wave velocity of the nerve to muscle (SWV_NMR_). *SD* standard deviation, *CV* coefficient variance, *a.u* arbitrary unit. *p < 0.05, **p < 0.01.

For the CSA and NBF values of the sciatic nerve, no significant difference was observed after nerve block in both the nerve-block and sham-block groups (Fig. 3a,b). In addition, for both values, there was no significant change in the values between the nerve-block and sham-block groups at any of the time points.

The SWV_N_ in the nerve-block group tended to increase from 30 to 90 min after the nerve block, although statistical analysis revealed no significant difference compared to the pre-block value (Fig. 3c). In the sham-block group, there was no obvious change in the SWV_N_ associated with the nerve block. The SWV_N_ values in the nerve-block group were significantly higher than those in the sham-block group at 90 min after the block (*p* = 0.040). In the SWV_M_, no obvious change associated with the nerve block was observed in the nerve-block and sham block groups (Fig. 3d). The SWV_NMR_ in the nerve-block group increased significantly from 30 min after the nerve block and remained high until 90 min; statistical analysis showed that there was a significant difference between the four time points (Kruskal–Wallis test, *p* = 0.002) and there were significant differences at each time point compared to the pre-block value (Steel–Dwass test, *p* = 0.020, *p* = 0.009, and *p* = 0.009, respectively) (Fig. 3e). There were significant differences in the SWV_NMR_ between the nerve-block and sham-block groups at 30, 60, and 90 min after the nerve block (*p* = 0.015, *p* = 0.007, and *p* = 0.003, respectively).

### Changes in the change rate of US parameters associated with the nerve block

**Figure 4:**
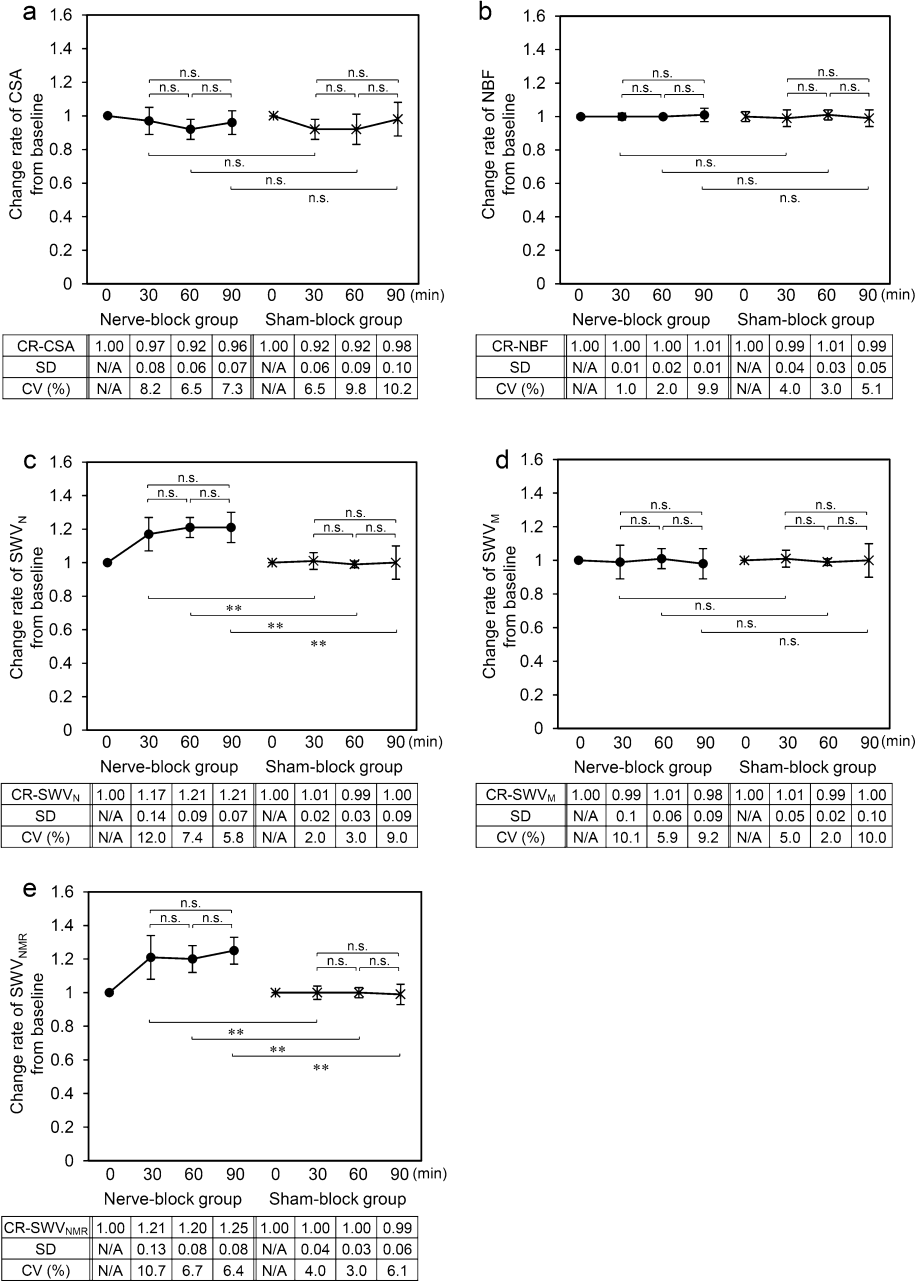
The change in the change rate of US parameters from the baseline value associated with the nerve block. (**a**) Change rate (CR) of cross-sectional area (CSA). (**b**) CR of nerve blood flow (NBF). (**c**) CR of shear wave velocity of the sciatic nerve (SWV_N_). (**d**) CR of shear wave velocity of the muscle (SWV_M_). (**e**) CR of the ratio of shear wave velocity of the nerve to muscle (SWV_NMR_). *SD* standard deviation, *CV* coefficient variance, *N/A* not applicable. *p < 0.05, **p < 0.01.

No significant difference exists in the change rates of the CSA or NBF between the nerve- and sham-block groups at any of the time points (Fig. 4a,b).

The change rate in the SWV_N_ from the baseline in the nerve-block group was significantly higher than that in the sham-block group at each time point (*p* = 0.001, *p* = 0.004, and *p* = 0.001, respectively) (Fig. 4c). In the change rate of SWV_M_, no significant difference between the nerve-block group and the sham-block group was observed (Fig. 4d). The change rate in the SWV_NMR_ in the nerve-block group was significantly higher than that in the sham-block group at each time point (*p* = 0.001, *p* = 0.001, and *p* = 0.001, respectively) (Fig. 4e).

### Diagnostic performance of SWV parameters for determining nerve block success

The above results indicate that the SWV_N_, SWV_NMR_, change rate in SWV_N_, and change rate in SWV_NMR_ could be indicators of the success of a nerve block. To clarify the diagnostic performance of these parameters for determining the success of a nerve block, we performed an ROC curve analysis. The results are summarized in Table 3.

**Table 3:** Sensitivity, specificity, and positive and negative predictive values according to cutoff values.

	SWV_N_	SWV_NMR_	Change rate in the SWV_N_	Change rate in the SWV_NMR_
Cutoff value	1.54	1.09	1.08	1.10
Sensitivity (%)	90.5 (69.6–98.8)	95.2 (76.2–99.9)	100 (77.2–100)	100 (77.2–100)
Specificity (%)	57.1 (39.4–73.7)	85.7 (69.7–95.2)	95.2 (76.2–100)	100 (77.2–100)
PPV (%)	55.9 (37.9–72.8)	80.0 (59.3–93.2)	95.5 (77.2–99.9)	100 (77.2–100)
NPV (%)	90.9 (70.8–98.9)	96.8 (83.3–99.9)	100 (76.2–100)	100 (77.2–100)
AUC	0.779 (0.658–0.900)	0.939 (0.883–0.996)	0.977 (0.932–1.000)	1.000 (1.000–1.000)
*p*-value versus SWV_N_	N/A	0.001**	0.008**	0.002**
*p*-value versus SWV_NMR_	N/A	N/A	0.064	0.051
*p*-value versus change rate in the SWV_N_	N/A	N/A	N/A	0.480

*PPV* positive predictive value, *NPV* negative predictive value, *AUC* area under the receiver operating characteristic curve. Numbers in parentheses are 95% confidence Intervals. *SWV*_*N*_ shear wave velocity of the sciatic nerve, *SWV*_*NMR*_ the ratio of shear wave velocity of the nerve to muscle, *N/A* not applicable, **p < 0.01.

The area under the curve (AUC) for the SWV_N_ was 0.779 with a cut-off value of 1.54, which are consistent with a moderate diagnostic accuracy^44^. The AUCs for SWV_NMR_, the change rate of SWV_N_, and the change rate in the SWV_NMR_ were 0.947, 0.998, and 1.000 when setting cut-off value of 1.09, 1.08, and 1.10, respectively, which are consistent with a high diagnostic accuracy. Notably, the sensitivity and specificity of the change rate in SWV_NMR_ were both 100%. The statistical analyses revealed that the AUC for SWV_N_ was significantly lower than those for the SWV_NMR_, the change rate in SWV_N_, and the change rate in the SWV_NMR_ (*p* = 0.001, *p* = 0.008, and *p* = 0.002, respectively). On the other hand, there was no significant difference between the AUC for the SWV_NMR_ and the change rate in SWV_N_, between the SWV_NMR_ and the change rate in the SWV_NMR_, or between the SWV_N_ change rate and SWV_NMR_ change rate.

## Discussion

Among the various ultrasonographic measurements studied herein, we observed increases in the SWV values of the nerve and the ratio of SWV values of the nerve to muscle in association with the nerve block. Our findings also demonstrated the high diagnostic performance of these SWV values for detecting the success of the nerve block, suggesting that SWV measurements of nerve and nerve/muscle ratio could be used as indicators of the success of regional nerve blocks. To the best of our knowledge, this is the first study to investigate the potential use of ultrasonographic parameters to identify the quality of regional nerve blocks.

The results of this study show that successful nerve blocks are associated with an increase in the SWV_N_ value compared with the pre-block value, beginning from 30 min after the injection of the local anesthetic and remaining for 90 min. Despite the lack of statistical significance, we strongly speculate that the increase in the SWV_N_ value is directly related to the nerve blockade rather than the serum levels of the local anesthetic or a secondary change due to the liquid injection and electric nerve stimulation, based on the following observations: (1) there was no obvious change in the value of SWV_N_ in the sham-block group, (2) the SWV_N_ values at 90 min post-block in the nerve-block group were significantly higher than those in the sham-block group, and (3) we assigned the legs of the same dog to the sham-block and nerve-block groups. Moreover, the change rate of the SWV_N_ in the nerve-block group was significantly higher than that in the sham-block group at all time points. The lack of a statistically significant difference in actual SWV_N_ values is probably due to fluctuations in this value among the individual dogs. Indeed, the CV values of the actual SWV_N_ were relatively high (> 10%) at all time points in both the nerve-block and sham-block groups. It has been demonstrated that the tissue elasticity of the peripheral nerve changes with various factors, including limb position and movement, laterality, and age^37–41^. On measuring the SWV of the dogs in prone position, it is possible that each dog had a different limb position, which may have affected this result.

In the present study, SWV_NMR_ significantly increased after nerve block and showed a higher diagnostic performance for detecting the success of the nerve block compared to SWV_N_. Since the CV values of SWV_NMR_ were lower than those of SWV_N_ and there was no obvious change in SWV_M_ from baseline in both the nerve-block and sham-block groups, we speculated the high diagnostic ability of SWV_NMR_ due to the normalization of SWV variation among individuals by calculating the nerve/muscle ratio in each individual. In SWE, differences in the measured values might have occurred due to the degree of probe compression even if the operator pays careful attention. Additionally, previous studies have reported that the SWE values were influenced by the depth of target regions^45^. To reduce the measurement variation due to these factors, it has been proposed to evaluate the ratio of the SWE value of the target tissue to that of the surrounding non target tissue^46,47^. Our results are consistent with these reports.

Our data also showed that the diagnostic performances of the change rates of SWV_N_ and SWV_NMR_ were higher than those of the actual measured values. Notably, the diagnostic accuracy of the change rate of SWV_NMR_ for successful nerve block was 100%, which was the highest among all the examined ultrasonic parameters. These change rates of SWV_N_ and SWV_NMR_ showing higher diagnostic abilities may be attributed to further normalization of the differences in SWVs between individuals compared with the actual SWV_N_ and SWV_NMR_ values through ratio calculations before and after nerve block This speculation is strongly supported by the results that the CV values of the change rates of SWV_N_ and SWV_NMR_ were lower than those of the respective actual measurements. Similar to our results, the findings of a previous study demonstrated that the relative change rate of the peripheral blood flow index from the baseline values has a higher diagnostic ability than actual values in determining the effect of the peripheral nerve block because of the large individual variation in baseline value^48^. Collectively, it is suggested that evaluating the ratio of SWV values of nerve to muscle and change rate in the SWV value, rather than measuring the actual SWV value itself, is an important factor for assessing nerve block success.

We observed no significant change in CSA in association with the nerve block. This result is consistent with a study that demonstrated that there was no morphological change in the sciatic nerve due to the continuous administration of local anesthetics in a rat model^32^. The small diameter of the sciatic nerve of dogs, that is, ≤ 2 mm, may also have affected this result. In addition to CSA, our results revealed no significant change in the NBF of the sciatic nerve in association with the nerve block, contrary to our expectations. Other investigations have demonstrated that local anesthetics, including levobupivacaine, ropivacaine, and bupivacaine, induce a reduction in nerve blood flow in animal models^32,33^. The discrepancy between these studies and our present results may be explained by differences in the measurement methods used. In previous studies, nerve blood flow was directly measured adjacent to the target nerve using a laser Doppler flowmeter^32,33^, which theoretically has a higher detection sensitivity of blood flow compared to SMI. We speculate that the change in the nerve blood flow induced by the local anesthetic is very faint and lower than the detection sensitivity of SMI, and as a result, the SMI failed to identify this blood flow change in the present study. Although the possibility may remain that SMI would detect the change in blood flow in human nerves (with larger diameters compared to dogs), our data suggest that the evaluation of nerve blood flow by SMI may be insufficient as an indicator of nerve block success. Additionally, the fact that the quantification of nerve blood flow by SMI is more complicated without the dedicated software compared to other examinations such as CSA and SWE is a considerable disadvantage of SMI for routine clinical applications.

The mechanism underlying the change in SWV values within the nerve associated with a nerve block is unclear, but several physiological mechanisms have been considered. The decrease in intraneural blood flow, reduction in the metabolic rate, and cytotoxic effect of local anesthetics may affect the change in SWV values. A reduction in blood flow within the nerve after the administration of local anesthetics has been demonstrated, and Crosby^48^ established that local anesthetics reduce the metabolic rate of the spinal cord, probably as a result of the profound sensory and motor block after spinal application. In addition, local anesthetics have various cytotoxic effects in cell cultures, including inhibition of cell growth, motility, and survival^49–51^. This may not apply directly to a peripheral nerve block, as these results are from experimental studies; however, it can be speculated that the combination of these effects causes transient ischemia and edema within the nerve, which finally leads to an increase in nerve stiffness. In support of this hypothesis, a relationship between nerve ischemia and an increase in nerve elasticity has been reported in diabetic neuropathy and compressive neuropathy^15,16,19,20^. Other direct and/or indirect anesthetic effects, such as the membrane-expanding effect of local anesthetics^52^ and the change in the elasticity of the surrounding tissues induced by a nerve block may also affect the change in the SWV values of a nerve.

An assessment using ultrasonography has several advantages over the currently used block assessment techniques. For example, US measurements, particularly an examination of SWE, are an objective means of assessing the outcome of a nerve block, unlike both the pinprick and cold sensation techniques, which require patients to report the precise sensation felt upon the application of a given stimulus. With US measurements, the patients were not subjected to the potential discomfort of a pinprick or icepack test. The US method can also be applied to patients undergoing general anesthesia. In addition, since US is used for nerve block, a one-stop assessment is possible. However, there are some drawbacks of an assessment using US elastography compared to the currently used block assessment techniques. The US method requires dedicated equipment. The measurement of the SWV is also relatively complex compared to the pinprick and cold sensation techniques.

This study has several limitations. First, the sample size was small, which may limit the power of the assessment of the efficacy of nerve block success by US measurements. Second, US elastography examination is limited by several technical difficulties, including the depth of the lesion and the proficiency of the operator, which limit its clinical application. Third, we did not directly assess sensory nerve block using a skin sensation test. However, this would not affect the outcome of the present experiment because the effect of a nerve block occurs on the sensory nerve, followed by the motor nerve^53^; thus, if the motor nerve block is achieved, it can be interpreted that the sensory-nerve block is also achieved. Forth, we evaluated the US measurements only from 30 min after the nerve block, and thus, we cannot make a definitive conclusion whether the SWV values can be a reliable predictor of the success of a nerve block at early time points after a nerve block. Finally, this was a preliminary animal study, and thus our results cannot be directly applied to actual human patients. In addition, the threshold of SWV value for predicting the success of nerve block might vary depending on the target nerves and injected local anesthetic agents. Further studies are needed to determine the applicability and reliability of SWV values to assess the success of a nerve block.

## Conclusions

The SWV values of the nerve and nerve/muscle ratio are observed to increase with nerve block. Ultrasonographic evaluations of these parameters could therefore be used as indicators having objective, noninvasive, and high diagnostic accuracies for predicting nerve block success.

## Data Availability

The datasets analysed during the current study are available from the corresponding author on reasonable request.
